# Health care resource utilization of patients with asthma and food allergy initiating omalizumab

**DOI:** 10.1016/j.jacig.2025.100491

**Published:** 2025-05-07

**Authors:** John A. Bird, Aimee M. Near, Julie Wang, Arpamas Seetasith, Vincent Garmo, Stella Ko, Xiaohui Zhao, Riddhi Doshi, Elizabeth J. Wang, Sachin Gupta, David M. Fleischer

**Affiliations:** a UT Southwestern Medical Center, Dallas, Tex; b IQVIA, Durham, NC; c Icahn School of Medicine at Mount Sinai, New York, NY; d Genentech, Inc, South San Francisco, Calif; e Children’s Hospital Colorado, University of Colorado, School of Medicine, Aurora, Colo

**Keywords:** Food allergy, asthma, health care resource use, health care costs, omalizumab, anaphylaxis, peanut allergy, emergency department visits, hospitalization

## Abstract

**Background:**

Patients with comorbid asthma and food allergy (FA) are at increased risk of adverse outcomes for both conditions. Omalizumab, a biologic for moderate to severe persistent asthma, received recent approval for FA.

**Objective:**

We sought to compare FA-, asthma/FA-related, and all-cause health care resource utilization and costs before and after omalizumab initiation among patients with asthma and FA.

**Methods:**

This study retrospectively analyzed data from IQVIA PharMetrics Plus claims among patients who were 6 years or older, had asthma and FA (medical claim for FA or anaphylactic reaction due to food), had initiated omalizumab between 2018 and 2021, and had 12 months of data before (baseline) and after (follow-up) omalizumab initiation (index date).

**Results:**

Of 523 patients who initiated omalizumab (mean age, 33.1 years; 71.5% female), the prevalence of FA-related (12.4% vs 4.8%), asthma/FA-related (25.0% vs 15.5%), and all-cause (44.6% vs 30.2%; all *P* < .0001) emergency department visits reduced at follow-up versus baseline. Significant reductions were also observed for the prevalence of hospitalizations (FA: 1.5% vs 0.2%; asthma/FA: 12.3% vs 7.4%; all-cause: 13.2% vs 7.5%) and total FA-related health care costs per patient ($1600 vs $1502; all *P* < .0001). The total asthma/FA-related ($5,966 vs $5,683, *P* = .8375) and all-cause ($24,874 vs $27,298, *P* = .6724) health care costs were similar from baseline to follow-up after excluding the omalizumab costs.

**Conclusions:**

FA-related, asthma/FA-related, and all-cause emergency department visits and hospitalizations significantly reduced in the 12 months after omalizumab initiation. These results provide valuable and timely real-world evidence that omalizumab has the potential to reduce the acute care utilization in patients with comorbid asthma and FA.

About 8% of children and 11% of adults are affected by food allergy (FA) in the United States.^1^^,^^2^ An increasing amount of research indicates that asthma and FA are highly correlated and are key components of the development of allergic diseases over the course of infancy and childhood, also known as the “atopic march.”^3^ Adults with asthma were found to be 1.9 times more likely to have FA compared with those without asthma,^4^ and children with FA had 3.3 to 3.7 times the risk of developing asthma compared with those without FA.^5^ Patients with both asthma and FA are at increased risk of adverse outcomes for both conditions.^6^ The severity of asthma is directly associated with the risk of anaphylaxis, which is tripled for patients with severe asthma.^7^ Among patients with asthma, FA has been associated with increased risk of life-threatening asthmatic episodes and increased health care resource use (HRU), including hospitalizations and emergency department (ED) visits.^4^^,^^8^

Management guidelines for FA emphasize the need for dietary avoidance of specific allergens and recommend prompt administration of intramuscular epinephrine for acute, life-threatening allergic food reactions.^9^, ^10^, ^11^ Omalizumab is a novel, recombinant, humanized, mAb against human IgE. Omalizumab had been approved by the US Food and Drug Administration (FDA) for several atopic conditions, including moderate to severe persistent asthma among adults and children aged 6 years or more.^12^ In February 2024, the US FDA approved omalizumab for the reduction of allergic food reactions, including anaphylaxis, that may occur with accidental exposure to 1 or more foods in adults and children aged 1 year or more.^12^ The approval was based on results from a phase 3 clinical trial (Omalizumab as Monotherapy and as Adjunct Therapy on Multi-Allergen Oral Immunotherapy [OIT] in Food Allergic Children and Adults [OUtMATCH]), indicating that omalizumab treatment for 16 to 20 weeks was superior to placebo in increasing the reaction threshold for multiple food.^13^

Although the efficacy and safety of omalizumab in the asthma/FA overlap population for reducing allergic reactions has been demonstrated in previous studies,^13^, ^14^, ^15^, ^16^, ^17^, ^18^, ^19^, ^20^ the real-world impact of omalizumab on FA- and asthma/FA-related HRU and costs in the United States remains unknown. To that end, this retrospective database study aimed to compare FA-, asthma/FA-related, and all-cause HRU and associated patient costs before and after initiation of omalizumab among patients with both asthma and FA in the United States.

## Methods

### Data source

This retrospective cohort study analyzed IQVIA PharMetrics Plus claims data between January 1, 2017, and December 31, 2022 (study period, which is before the FDA approval of omalizumab for FA). PharMetrics Plus is a health insurance plan claims database composed of fully adjudicated medical and pharmacy claims of commercial as well as managed Medicare and Medicaid plans for more than 210 million unique enrollees. It is representative of the commercially insured US population for patients younger than 65 years. In compliance with the Health Insurance Portability and Accountability Act, patient data included in the analyses were deidentified; therefore, an institutional review board review was not required for the study.

### Study design and patient selection

**Fig 1:**
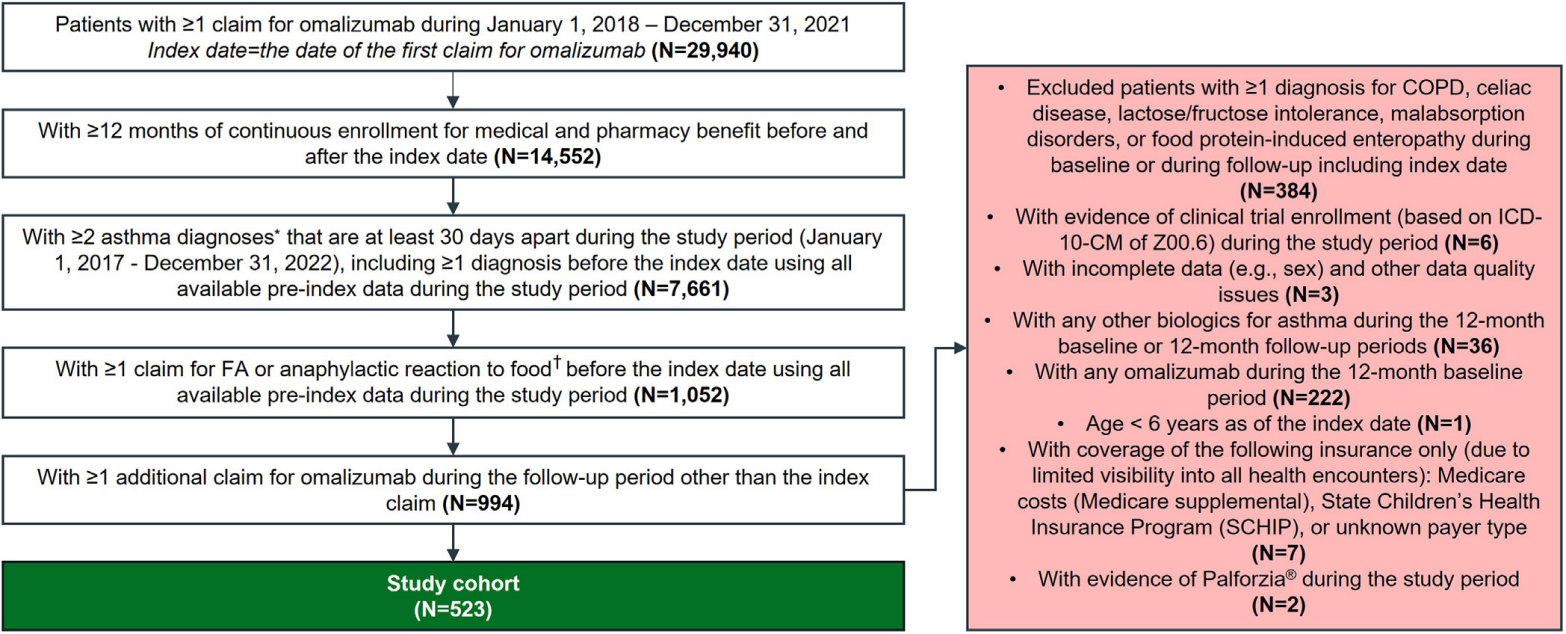
Stepwise attrition of the study cohort. ∗Asthma was identified by *ICD-10-CM* codes of J45.X and J82.83. †FA was identified by *ICD-10-CM* codes of Z91.01X; anaphylactic reaction to food was identified by *ICD-10-CM* codes of T78.0X excluding T78.06X. *COPD*, Chronic obstructive pulmonary disease; *ICD-10-CM*, *International Classification of Diseases, Tenth Revision, Clinical Modification*.

The patient selection process is illustrated in Fig 1. Briefly, patients aged 6 years or older with asthma (patients were assumed to have moderate to severe asthma per the US FDA-approved indication for omalizumab at the time of the analysis^12^) and documented FA based on the *International Classification of Diseases, Tenth Revision, Clinical Modification* diagnosis codes (see Table E1 in this article’s Online Repository at www.jaci-global.org) who newly initiated omalizumab between January 1, 2018, and December 31, 2021 (selection window), were identified. The date of the first omalizumab claim during the selection window was termed as the index date. All patients were required to have continuous health plan enrollment for medical and pharmacy benefits for at least 12 months before (baseline period) and after (follow-up period) the index date as well as 1 or more additional claim for omalizumab after the index date during the follow-up period. To minimize confounding and misclassification, patients with 1 or more diagnosis code for chronic obstructive pulmonary disease, celiac disease, lactose/fructose intolerance, malabsorption disorders, or food protein–induced enteropathy and those with any claims of other biologics for asthma (ie, mepolizumab, reslizumab, benralizumab, dupilumab, tezepelumab-ekko) during the 12-month baseline or follow-up period were excluded.

### Study measures

Patients’ demographic characteristics were assessed on the basis of data on the index date or date closest to and before the index date during the 12-month baseline period. Baseline clinical characteristics, including the type of FA (single or multiple FA, allergy to food, including unspecified FA based on available *International Classification of Diseases, Tenth Revision, Clinical Modification* diagnosis codes; see Table E1), Charlson comorbidity index (modified Quan adaptation) excluding asthma,^21^ select non–Charlson comorbidity index conditions, documented anaphylactic reaction to food, and prior epinephrine injection in a health care setting (ie, hospital, ED, or outpatient clinic) were assessed during the 12-month baseline period.

FA-, asthma/FA-related, and all-cause HRU and associated costs were evaluated during the 12-month baseline and follow-up (including index date) periods. FA-related HRU and costs were assessed using claims with a diagnosis for FA, anaphylactic reaction to food, or other adverse food reactions (see Table E1) in the primary position for hospitalizations and in any position for other medical services. Because of the potential overlap in the characterization of symptoms of an asthma exacerbation and allergic reactions to food due to accidental exposure,^22^ composite measures for asthma/FA-related HRU and costs were examined using claims with a diagnosis code for FA or asthma.

HRU, including ED visits that did not lead to hospitalizations, hospitalizations, and outpatient physician office visits, was quantified by the proportion of patients with 1 or more corresponding event and the number of such events (on distinct dates) per patient. Cost measures included costs for each utilization category described above, total health care costs for hospitalizations, ED visits, outpatient physician office visits, office-administered medication (identified by Healthcare Common Procedure Coding System codes), other medical services, and outpatient pharmacy (identified by National Drug Code), and total health care costs excluding the costs of omalizumab (nonomalizumab costs). Nonomalizumab FA-related costs were not evaluated because omalizumab was not approved for FA alone at the time of the analysis. All costs were adjusted to 2023 US dollars using the medical care component of the Consumer Price Index.

### Statistical analyses

All study measures were analyzed descriptively and presented as frequencies with percentages for binary/categorical variables and means (with SD) or median (with interquartile range) for continuous variables. HRU and cost measures during the 12-month baseline and follow-up periods were compared using the McNemar test for the proportion of patients with 1 or more event, zero-inflated negative binomial regression for the number of events per patient, and the Wilcoxon signed-rank test for costs. Health care costs were determined using the “allowed” amount field, which represents the reimbursed amount paid by payers combined with the patient out-of-pocket cost (eg, co-pay and co-insurance). As a sensitivity analysis, HRU and associated costs before and after omalizumab initiation were compared in a subgroup of patients who were likely persistent to omalizumab (ie, received at least 6 doses of omalizumab during the 12-month follow-up period, assuming an average gap between consecutive doses was no longer than 2 times of the longest dosing interval [8 weeks] for asthma per the US FDA label^12^). All data management and analyses were performed using the software SAS version 9.4 (SAS Institute, Inc, Cary, NC).

## Results

### Study cohort and baseline characteristics

The study cohort included 523 patients with asthma and FA who initiated omalizumab between 2018 and 2021 (Fig 1). Patients’ baseline characteristics are presented in Table I. In summary, patients had a mean age of 33.1 ± 16.7 years (24.5% were younger than 18 years) and were predominantly female (71.5%). During the 12-month baseline period, most patients had a Charlson comorbidity index of 0 (76.7%). Allergic rhinitis (83.0%) was the most common comorbidity observed. Most patients had unspecified FA (no *International Classification of Diseases, Tenth Revision,Clinical Modification* diagnosis codes for known type of FA, 65.6%), 15.7% had documented single FA (presence of diagnosis codes for only 1 known FA without any diagnosis codes for unspecified FA), and 8.2% had documented multiple FAs (presence of diagnosis codes for more than 1 known FA with or without any codes for unspecified FA). Seafood allergies (15.7%) were the most prevalent specified FAs, followed by peanut allergies (9.8%). Approximately 27.5% had documented anaphylactic reaction to food, and 7.8% had 1 or more epinephrine injection at a health care setting during the baseline period (Table I).

**Table I:** Demographic and baseline clinical characteristics of patients with asthma and FA initiating omalizumab

Demographic and clinical characteristics∗	Study cohort (N = 523)
Age (y)	
Mean ± SD	33.1 ± 16.7
Median (Q1, Q3)	33.0 (18.0, 47.0)
<18 y	128 (24.5)
Sex	
Female	374 (71.5)
Male	149 (28.5)
Geographic region	
Northeast	92 (17.6)
Midwest	128 (24.5)
South	225 (43.0)
West	78 (14.9)
Payer type	
Third-party	329 (62.9)
Self-insured	188 (35.9)
Managed Medicare	3 (0.6)
Managed Medicaid	3 (0.6)
Index year	
2018	120 (22.9)
2019	138 (26.4)
2020	133 (25.4)
2021	132 (25.2)
CCI†	
Mean ± SD	0.4 ± 1.0
Median (Q1, Q3)	0.0 (0.0, 0.0)
CCI categories†	
0	401 (76.7)
1	75 (14.3)
2+	31 (5.9)
Respiratory comorbidities	
Chronic sinusitis	97 (18.5)
Obstructive sleep apnea	41 (7.8)
Pneumonia	29 (5.5)
COVID-19	18 (3.4)
Allergy-related comorbidities	
Allergic rhinitis	434 (83.0)
Eczema/atopic dermatitis	82 (15.7)
Angioedema	77 (14.7)
Allergic conjunctivitis	70 (13.4)
Allergic urticaria	70 (13.4)
Patients with documented single FA‡	82 (15.7)
Patients with documented multiple FAs§	43 (8.2)
Patients with documented anaphylactic reaction to food||	144 (27.5)
Patients with ≥1 epinephrine injection in hospital/ED/outpatient office¶	41 (7.8)

Values are n (%) unless otherwise indicated.

*CCI*, Charlson comorbidity index; *COVID-19*, coronavirus disease 2019; *HCPCS*, Healthcare Common Procedure Coding System; *Q1*, first quartile; *Q3*, third quartile.

∗ Demographic characteristics were assessed on or closest before the date of the first omalizumab prescription (index date). Clinical characteristics were assessed during the 12 mo before the index date.

† CCI was assessed using a modification of Quan adaptation wherein asthma was not included in the assessment of CCI because all patients in the study population were required to have a diagnosis of asthma.

‡ Patients with documented single FA were identified by the presence of only 1 known FA without unspecified FA based on *ICD-10-CM* diagnosis codes.

§ Patients with documented multiple FAs were identified by the presence of >1 known FA with or without unspecified FA based on *ICD-10-CM* diagnosis codes.

|| Anaphylactic reaction to food was identified by *ICD-10-CM* codes of T78.0X (except T78.06X anaphylactic reaction to food additives) and T78.1.

¶ Epinephrine injections administered in hospital or office settings were identified by HCPCS codes.

### ED visits and associated costs

**Fig 2:**
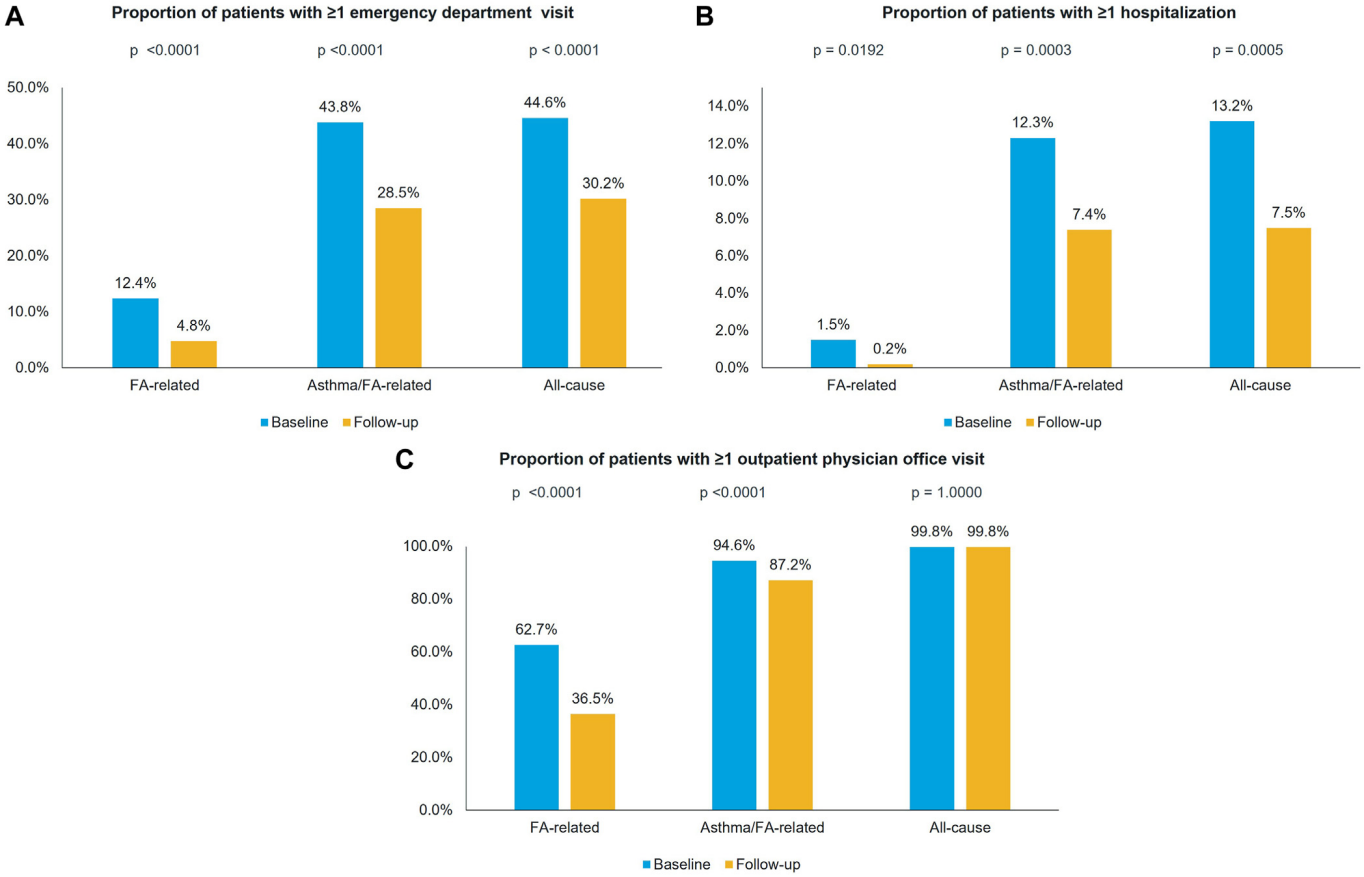
FA-related, asthma/FA-related, and all-cause HRU during the 12-month baseline and follow-up periods. **A,** Proportion of patients with at least 1 ED visit. **B,** Proporiton of patients with at least 1 hospitalization. **C,** Proportion of patients with at least 1 outpatient physician office visit. FA-related hospitalizations were identified by a diagnosis for FA (Z91.01X), anaphylactic reaction to food (T78.0X excluding T78.06X), or other adverse food reactions (T78.1) in the primary position of the claims. FA-related ED visits and outpatient physician office visits were identified by a diagnosis for FA, anaphylactic reaction to food, or other adverse food reactions in any position of the claims. Asthma/FA-related hospitalizations were identified by a diagnosis for asthma (J45.X and J82.83), FA (Z91.01X), anaphylactic reaction to food (T78.0X excluding T78.06X), or other adverse food reactions (T78.1) in the primary position of the claims. Asthma/FA-related ED visits and outpatient physician office visits were identified by a diagnosis for asthma, FA, anaphylactic reaction to food, or other adverse food reactions in any position of the claims.

The proportions of patients with 1 or more FA-related (baseline: 12.4% vs follow-up: 4.8%), 1 or more asthma/FA-related (25.0% vs 15.5%), and 1 or more all-cause (44.6% vs 30.2%; all *P* < .0001) ED visit were significantly lower during the follow-up period (Fig 2, *A*). A statistically significant decrease was also observed for the mean number of all-cause ED visits per patient (1.2 vs 0.7, *P* = .0418). The mean numbers of FA- (0.2 vs 0.1, *P* = .2553) and asthma/FA-related (0.5 vs 0.3, *P* = .2206) ED visits per patient were numerically lower during follow-up.

In terms of health care costs associated with ED visits, the per-patient costs for FA-related ($183 vs $85), asthma/FA-related ($636 vs $375), and all-cause ($1530 vs $918; all *P* < .01) ED visits decreased in the follow-up period (Table II).

**Table II:** FA-related, asthma/FA-related, and all-cause health care costs (in 2023 USD) in the 12 mo before (baseline) and after (follow-up) omalizumab initiation among patients with asthma and FA

Cost categories	Baseline mean ± SD costs	Follow-up mean ± SD costs	*P* value
FA-related∗			
Hospitalization	$242 ± $3,614	$45 ± $1,023	.0977
ED visit	$183 ± $826	$85 ± $566	.0002
Outpatient physician office visit	$320 ± $921	$224 ± $880	<.0001
Total†	$1,600 ± $4,761	$1,502 ± $5,253	<.0001
Asthma/FA-related‡			
Hospitalization	$1,082 ± $5,720	$115 ± $1,346	<.0001
ED visit	$636 ± $2,454	$375 ± $1,427	.0003
Outpatient physician office visit	$878 ± $1,201	$750 ± $1,640	<.0001
Total†	$5,966 ± $9,692	$34,927 ± $23,239	<.0001
Total nonomalizumab§	$5,966 ± $9,692	$5,683 ± $9,811	.8375
All-cause			
Hospitalization	$4,359 ± $20,517	$5,250 ± $44,124	.0036
ED visit	$1,530 ± $4,432	$918 ± $2,615	<.0001
Outpatient physician office visit	$3,584 ± $3,485	$3,658 ± $4,163	.1081
Total	$24,874 ± $43,020	$56,542 ± $62,918	<.0001
Total non-omalizumab§	$24,874 ± $43,020	$27,298 ± $60,068	.6724

*USD*, United States dollars.

∗ FA-related costs were identified by a diagnosis for FA (Z91.01X), anaphylactic reaction to food (T78.0X excluding T78.06X), or other adverse food reactions (T78.1) in the primary position of the claims for hospitalizations and in any position of the claims for ED visits, outpatient physician office visits, and other medical services.

† Total health care costs included costs for ED visits that did not lead to hospitalizations, hospitalizations, outpatient physician office visits, other medical services, and outpatient pharmacy.

‡ Asthma/FA-related costs was identified by a diagnosis for asthma (J45.X and J82.83), FA (Z91.01X), anaphylactic reaction to food (T78.0X excluding T78.06X), or other adverse food reactions (T78.1) in the primary position of the claims for hospitalizations and in any position of the claims for ED visits, outpatient physician office visits, and other medical services.

§ Total nonomalizumab costs were estimated as the total health care costs, excluding the costs of omalizumab. Total FA-related nonomalizumab costs were not estimated in the study because omalizumab had not been approved for FA during the study analysis.

### Hospitalizations and associated costs

There were significant reductions in the proportions of patients with 1 or more FA-related (1.5% vs 0.2%, *P* < .0001), asthma/FA-related (12.3% vs 7.4%, *P* < .0001), and all-cause (13.2% vs 7.5%, *P* < .0001) hospitalization in the 12 months after omalizumab initiation compared with preinitiation (Fig 2, *B*). The mean numbers of FA-related (0.02 vs 0.00, *P* = .0357) and asthma/FA-related hospitalizations (0.1 vs 0.0, *P* = .0094) per patient were also significantly lower during follow-up. The reduction in the mean number of all-cause hospitalizations (0.2 vs 0.1, *P* = .0994) was not statistically significant.

During the follow-up period, there was a significant reduction in the mean per-patient costs that were associated with asthma/FA-related hospitalizations ($1082 vs $115, *P* < .0001). The mean costs for FA-related hospitalizations were numerically lower during the follow-up period ($242 vs $45, *P* = .0977). The mean all-cause hospitalization cost ($4359 vs $5250, *P* = .0036) per patient was higher during the follow-up than during the baseline period (Table II).

### Outpatient physician office visits and associated costs

In the 12 months after omalizumab initiation, there was a significant decrease in the proportions of patients with 1 or more FA- (62.7% vs 36.5%) and asthma/FA-related (94.6% vs 87.2%; both *P* < .0001) outpatient physician office visit. The proportion of patients with 1 or more all-cause outpatient physician visit was similar between baseline and follow-up (99.8% vs 99.8%, *P* = 1.0000; Fig 2, *C*). Significant reductions were also observed for the mean number of FA-related outpatient physician office visits per patient (1.7 vs 1.4, *P* = .0018) as well as the mean per-patient costs for FA- ($320 vs $224, *P* < .0001) and asthma/FA-related ($878 vs $750, *P* < .0001) outpatient physician office visits. The mean per-patient costs for all-cause outpatient physician office visits were similar between the baseline and follow-up periods ($3584 vs $3658, *P* = .1081; Table II).

### Total health care and nonomalizumab costs

During the 12 months after omalizumab initiation, there was a statistically significant decrease in the total FA-related health care costs per patient ($1600 vs $1502, *P* < .0001). The mean (1) asthma/FA-related ($5,966 vs $34,927) and (2) all-cause ($24,874 vs $56,542; both *P* < .0001) total health care costs per patient (both categories included costs of omalizumab) were significantly higher during the follow-up; however, the total nonomalizumab costs were similar between the 2 time periods (asthma/FA-related: $5,966 vs $5,683, *P* = .8375; all-cause: $24,874 vs $27,298, *P* = .6724; Table II).

### Sensitivity analysis

A total of 432 patients received 6 or more omalizumab doses during the 12-month follow-up period and were included in the sensitivity analysis. Similar trends of HRU and cost changes from baseline to follow-up were observed (see Table E2 in this article’s Online Repository at www.jaci-global.org). Briefly, the utilization of FA-related, asthma/FA-related, and all-cause ED visits and hospitalizations as well as FA- and asthma/FA-related outpatient physician office visits decreased from the baseline to the follow-up period. Similar reductions were also observed for FA-related, asthma/FA-related, and all-cause ED costs, asthma/FA-related hospitalization costs, as well as total FA-related health care costs.

## Discussion

This is the first US real-world study evaluating the HRU and associated costs among patients with comorbid FA who received omalizumab. This study provides timely real-world evidence in a patient population that is at elevated risk of adverse outcomes for both asthma and FA by examining multiple measures of HRU and costs during the 12 months before and after omalizumab initiation. Overall, the study findings suggest that omalizumab may be effective in reducing FA-, asthma/FA-related, and all-cause ED visits and hospitalizations as well as total FA-related health care costs among patients with comorbid asthma and FA. Results of the OUtMATCH phase 3 randomized double-blind placebo-controlled trial demonstrate the efficacy of omalizumab in increasing the reaction threshold for peanut and other common food allergens to levels that are likely to exceed those that would be needed for the amounts of food that are typically encountered during accidental exposure^23^ and highlight the potential in using omalizumab as a preventative management option to reduce the daily risk of food allergic reactions. Our findings further add evidence to the OUtMATCH phase 3 trial^13^ that supports the benefits of omalizumab for FA.

Allergic reactions to food can be life-threatening and may require ED visits and hospitalizations, especially among children and teenagers, in those with concurrent asthma due to the risk of severe bronchospasm and anaphylaxis being greater.^24^ ED visits are common among patients with FAs, with 38.3% reporting 1 or more FA-related ED visit during their lifetime and 8.6% reporting 1 or more visit within the past year in a US population-based survey.^2^ Another study estimated that FA-related anaphylaxis results in 30,000 ED visits in the United States each year.^25^ Our study found a higher prevalence of FA-related ED visits in the year before omalizumab initiation (12.4%), likely due to the presence of comorbid asthma, which has been associated with increased risk of anaphylaxis.^7^ We also demonstrated a 14.4% reduction in the proportion of patients with all-cause ED visits during the 12 months following omalizumab initiation, with most (7.6%) of this from reduced asthma/FA-related ED visits. These reductions in ED visits correspond to $612 of savings per patient per year for the total costs of ED visits.

The US Centers for Disease Control and Prevention estimates nearly 10,000 FA-related hospitalizations annually among patients younger than 18 years.^24^ In this study, we found that prevalence of hospitalizations for FA and hospitalizations for asthma or FA reduced by 86.7% (1.5% to 0.2%) and 65.6% (6.1% to 2.1%) from baseline to follow-up, respectively. These results are in line with existing evidence suggesting that omalizumab reduces the incidence and frequency of asthma-related HRU, including ED visits and hospitalizations, among patients with moderate to severe asthma across age groups with concomitant FA.^14^^,^^26^ Although the mean costs per patient for FA- and asthma/FA-related hospitalizations decreased in the 12 months following omalizumab initiation, the mean all-cause hospitalization costs increased. *Post hoc* exploration suggests that this increase may have been primarily driven by a few patients with extreme long stays (eg, 1 patient was hospitalized for 109 days, whereas the median total length of stay for all-cause hospitalizations was 5 days among patients who had at least 1 hospitalization) in the follow-up period. Lastly, outpatient follow-up management is often needed following ED visits for anaphylaxis and, as expected, was common among patients in our study. Notably, the prevalence of FA-related outpatient physician office visits reduced by 41.8% (62.7% to 36.5%) during the follow-up. The reduction in FA- and asthma/FA-related visits and costs in the follow-up may be a combined result of reduced FA- and asthma/FA-related ED visits as well as reduced unscheduled office visits for asthma exacerbations.^27^

In terms of health care costs, although there were significant reductions in the costs for FA- and asthma/FA-related ED visits and hospitalizations in the follow-up period compared with baseline, the total health care costs increased due to the costs of omalizumab. Similar results were observed in the subgroup of patients who were likely persistent to omalizumab (ie, received at least 6 doses of omalizumab in the 12 months following initiation). Our findings demonstrate the benefits of omalizumab in reducing FA- and asthma/FA-related acute care and relevant costs among patients with comorbid asthma and FA. Ensuring access to omalizumab may benefit patient populations at risk of adverse outcomes (eg, ED visits or hospitalizations), including patients with comorbid asthma and FA by reducing the risk of severe bronchospasm and anaphylaxis. There is evidence showing that Black individuals had higher prevalence of moderate to severe asthma and FA and may be less likely to use asthma biologics compared with White individuals.^28^, ^29^, ^30^ Race/ethnicity is not captured by the study data and therefore was not examined in the study. Future studies exploring the benefit of omalizumab among Black patients with comorbid asthma and FA are warranted.

It is important to consider study limitations associated with the data source and study design when interpreting the results. Common limitations related to claims data, including possible billing and coding errors, could lead to potential misclassification of diagnoses and/or treatments. In particular, most patients only had unspecified FA recorded with diagnosis codes, and definitions of single FA and multiple FAs based on diagnosis codes for specific FA may not lead to an accurate categorization of these patient populations. Confirmatory information about the diagnosis of FA (including skin, blood, or food challenge testing) was not available. Similarly, utilization and costs for FA- and asthma/FA-related ED visits and outpatient medical services were identified by a relevant diagnosis code in any position of the claims and may include utilization and costs that were not primarily for asthma or FA. To that end, the asthma and/or FA-related outpatient medical utilization and costs could have been overestimated in the study.

It is important to note that the HRU and costs before and after omalizumab initiation were compared in a single cohort with no control group. The reductions in FA-related HRU and costs observed may be a result of the regression-to-the-mean effect^31^ and patient behavior changes due to evolving knowledge about FAs. Although a control group can help adjust for regression-to-the-mean effect and other confounding factors, it is difficult to identify a comparable control group without introducing other biases in claims data analysis. The single cohort pre-post design was used in this study to obtain signs of potential treatment effect to inform future studies. In addition, more than half of the study cohort had their follow-up period overlap with the coronavirus disease 2019 pandemic (ie, March 2020 to December 2022). The reduction in HRU and costs of these patients may partially be a result of reduced access to care and care-seeking during the pandemic, and therefore the impact of omalizumab on these outcomes may be overestimated. Lastly, the study results were based on analyses of health care claims data of patients who had FA and asthma documented in the claims data during the study period and therefore may not be generalizable to all patients with FA and asthma in the United States.

### Conclusions

This retrospective study compared HRU and costs before and following initiation of omalizumab among patients with both moderate to severe asthma and FA. There were significant reductions in utilization and costs in FA-related, asthma/FA-related, and/or all-cause ED visits and hospitalizations in the 12 months after omalizumab initiation. These reductions may indicate improved management of asthma and FA. Our study results provide valuable and timely evidence that omalizumab has the potential to reduce the acute care utilization in patients with comorbid asthma and FA.

**Clinical implications**

Omalizumab has the potential to reduce ED visits and hospitalizations among patients with comorbid asthma and FA.

## Disclosure statement

The study was funded by 10.13039/100004328Genentech, Inc, a member of the 10.13039/100004337Roche Group.

Disclosure of potential conflict of interest: J. A. Bird is a consultant for Allakos, AllerGenis, Allergy Therapeutics, Ltd, DBV Technologies, Food Allergy Research & Education (FARE), Genentech, HAL Allergy, Hanimmune Therapeutics, Novartis, Nutricia, and Parexel; and has received grant funding (institution) from Aimmune, DBV Technologies, FARE, Genentech, National Institutes of Health (NIH) National Institute of Allergy and Infectious Diseases, Novartis, Regeneron, and Siolta. E. J. Wang, R. Doshi, X. Zhao, and A. M. Near are employees of IQVIA, which received funding from Genentech, Inc, to conduct this study. D. M. Fleischer is a consultant for Aquestive, ARS Pharmaceuticals, Bryn Pharma, DBV Technologies, Genentech, and Nasus Pharma and a speaker for Genentech; has received grant funding to institution from ARS Pharmaceuticals and DBV Technologies; and has received stock options from Grow Happy. J. Wang reports clinical trials support (money to institution) from the NIH, Aimmune, DBV Technologies, and Siolta outside the submitted work; and consulting fees from DBV Technologies and Novartis outside the submitted work. A. Seetasith, S. Ko, V. Garmo, and S. Gupta are employees of Genentech, Inc, and stockholders of the Roche Group.
