# Supplementary material for: Health care resource utilization of patients with asthma and food allergy initiating omalizumab
Source: J Allergy Clin Immunol Glob. 2025 May 7;4(3):100491. doi: 10.1016/j.jacig.2025.100491 (PMC12151666; doi:10.1016/j.jacig.2025.100491)
Supplement: Supplementary Figs and Tables [file mmc1.docx]

**Supplemental material**

**Supplemental table I.** **ICD-10-CM diagnosis codes for identifying patients with asthma and FA^1^ and FA-related healthcare resource utilization.^2^**

| **Condition** | **ICD-10-CM codes** |
| --- | --- |
| Asthma | J45.X and J82.83 |
| FA | Z91.01X |
| Anaphylactic reaction to food | T78.0X excluding T78.06X |
| Other adverse food reactions | T78.1 |

**Notes:**

1. Patients with asthma and FA were identified using ICD-10-CM diagnosis codes for asthma, FA, and anaphylactic reaction to food.
2. FA-related healthcare resource utilization was identified using ICD-10-CM diagnosis codes for asthma, FA, anaphylactic reaction to food, and other adverse food reactions.

**Abbreviations:**

FA, food allergy; ICD-10-CM, International Classification of Diseases, tenth revision.

**Supplemental table II.** **FA-related, asthma/FA-related and all-cause healthcare resource utilization and costs in the 12 months before (baseline) and after (follow-up) omalizumab initiation among patients with asthma and FA who received ≥6 omalizumab doses (n=432) during the 12-month follow-up.**

|  | **Baseline** | **Follow-up** | **p-value^1^** |
| --- | --- | --- | --- |
| **FA-related^2^** | | | |
| Proportion of patients with ≥1 event (n, %) | | | |
| ED visit | 12.3% | 4.9% | <0.0001 |
| Hospitalization | 1.6% | 0.0% | NA |
| Outpatient physician office visit | 63.0% | 37.5% | <0.0001 |
| Mean (SD) number of events per patient | | | |
| ED visit | 0.2 (0.6) | 0.1 (0.5) | 0.3696 |
| Hospitalization | 0.0 (0.2) | 0.0 (0.0) | NA |
| Outpatient physician office visit | 1.8 (3.7) | 1.5 (4.4) | 0.0040 |
| Mean (SD) costs per patient (2023 USD) | | | |
| Hospitalization | $265 ($3,935) | $0 ($0) | 0.0156 |
| ED visit | $179 ($852) | $91 ($607) | 0.0037 |
| Outpatient physician office visit | $343 ($1,003) | $244 ($955 | <0.0001 |
| Total^3^ | $1,594 ($5,009) | $1,592 ($5,536) | <0.0001 |
| **Asthma/FA-related^4^** | | | |
| Proportion of patients with ≥1 event (n, %) | | | |
| ED visit | 24.8% | 15.5% | <0.0001 |
| Hospitalization | 6.0% | 1.6% | 0.0003 |
| Outpatient physician office visit | 94.0% | 88.0% | 0.0002 |
| Mean (SD) number of events per patient | | | |
| ED visit | 0.5 (1.2) | 0.3 (0.9) | 0.2098 |
| Hospitalization | 0.1 (0.4) | 0.0 (0.2) | 0.0023 |
| Outpatient physician office visit | 5.4 (5.6) | 5.7 (7.0) | 0.0865 |
| Mean (SD) costs per patient (2023 USD) | | | |
| Hospitalization | $1,071 ($5,917) | $62 ($901) | <0.0001 |
| ED visit | $643 ($2,614) | $391 ($1,497) | 0.0071 |
| Outpatient physician office visit | $918 ($1,280) | $801 ($1,766) | <0.0001 |
| Total^3^ | $6,025 ($9,773) | $39,376 ($22,864) | <0.0001 |
| Total non-omalizumab^5^ | $6,025 ($9,773) | $6,041 ($10,450) | 0.3360 |
| **All-cause** | | | |
| Proportion of patients with ≥1 event (n, %) | | | |
| ED visit | 43.8% | 28.5% | <0.0001 |
| Hospitalization | 12.3% | 7.4% | 0.0082 |
| Outpatient physician office visit | 99.8% | 99.8% | 1.0000 |
| Mean (SD) number of events per patient | | | |
| ED visit | 1.1 (2.3) | 0.6 (1.5) | 0.0317 |
| Hospitalization | 0.2 (0.8) | 0.1 (0.5) | 0.0735 |
| Outpatient physician office visit | 25.8 (23.7) | 28.2 (25.4) | 0.0951 |
| Mean (SD) costs per patient (2023 USD) | | | |
| Hospitalization | $4,507 ($22,178) | $5,551 ($47,387) | 0.0123 |
| ED visit | $1,521 ($4,433) | $864 ($2,494) | <0.0001 |
| Outpatient physician office visit | $3,542 ($3,537) | $3,685 ($4,233) | 0.4300 |
| Total^3^ | $25,449 ($45,990) | $61,097 ($66,195 | <0.0001 |
| Total non-omalizumab^5^ | $25,449 ($45,990) | $27,762 ($63,968 | 0.6520 |

**Notes:**

- - - 1. P-value was derived from McNemar test for proportions, zero-inflated negative binomial regression for number of events and Wilcoxon signed-rank tests for costs.
      2. FA-related costs were identified by a diagnosis for FA (Z91.01X), anaphylactic reaction to food (T78.0X excluding T78.06X), or other adverse food reactions (T78.1) in the primary position of the claims for hospitalizations and in any position of the claims for ED visits, outpatient physician office visits, and other medical services.
      3. Total healthcare costs included costs for ED visits that are not leading to hospitalizations, hospitalizations, outpatient physician office visits, other medical services, and outpatient pharmacy.
      4. Asthma/FA-related costs was identified by a diagnosis for asthma (J45.X and J82.83), FA (Z91.01X), anaphylactic reaction to food (T78.0X excluding T78.06X), or other adverse food reactions (T78.1) in the primary position of the claims for hospitalizations and in any position of the claims for ED visits, outpatient physician office visits, and other medical services.
      5. Total non-omalizumab costs were estimated as the total healthcare costs, excluding the costs of omalizumab. Total FA-related non-omalizumab costs were not estimated in the study as omalizumab had not been approved for FA during the study analysis.

**Abbreviations:** ED, Emergency department; FA, food allergy; NA, not available; SD, standard deviation; USD, United States Dollars.
